# The role of aryl hydrocarbon receptor signalling in COVID-19 pathology and its therapeutic potential

**DOI:** 10.3389/fmmed.2025.1599785

**Published:** 2025-08-29

**Authors:** Saidon Mbambara, Ndimo Modipane, Thato Serite, Mike Sathekge, Mankgopo Kgatle

**Affiliations:** ^1^ Department of Nuclear Medicine, University of Pretoria and Steve Biko Academic Hospital, Pretoria, South Africa; ^2^ Nuclear Medicine Research Infrastructure (NuMeRI), Department of Basic and Translational Research, Steve Biko Academic Hospital, Pretoria, South Africa; ^3^ Department of Biomedical Sciences, Tropical Diseases Research Centre, Ndola, Zambia; ^4^ Department of Medicine, University of Cape Town and Groote Schuur Hospital, Observatory, Cape Town, South Africa

**Keywords:** aryl hydrocarbon receptor, COVID-19, SARS-CoV-2, comorbidities, diabetes, hypertension, inflammation, metabolic disorders

## Abstract

Coronavirus disease 2019 (COVID-19), caused by the *betacoronavirus* SARS-CoV-2, emerged in Wuhan, China, and rapidly evolved into a global health crisis. Recent evidence highlights the activation of the aryl hydrocarbon receptor (AHR) pathway following SARS-CoV-2 infection, implicating AHR in facilitating viral replication and impairing antiviral immunity. As a ligand-dependent transcription factor, AHR regulates immune responses, cellular differentiation, and proliferation, and is frequently exploited by viruses to evade host defences. In relation to COVID-19, AHR activation drives immune suppression, systemic inflammation, and metabolic disturbances, intensifying disease severity. Notably, in individuals with comorbidities such as obesity and diabetes, AHR overactivity exacerbates insulin resistance, oxidative stress, endothelial dysfunction, and thrombotic risk, contributing to cardiovascular complications. AHR also promotes airway remodelling and mucus hypersecretion, fostering respiratory dysfunction and fibrotic progression. This review synthesizes current insights into the mechanistic role of AHR signalling in SARS-CoV-2 pathogenesis and discusses its potential as a target for host-directed therapeutic interventions.

## Introduction

Coronavirus disease 2019 (COVID-19) is an acute respiratory illness caused by severe acute respiratory syndrome coronavirus −2 (SARS-CoV-2), which first emerged in Wuhan, China, in December 2019 (Tang et al., 2020). SARS-CoV-2 is a positive-sense, single-stranded RNA virus belonging to the *betacoronavirus* genus within the *Coronaviridae* family (Hu et al., 2023). Transmission among humans primarily occurs via respiratory droplets and aerosols, with viral particles capable of persisting for hours to days depending on environmental conditions (Al et al., 2020; Brodin, 2021). Entry of the virus into host cells is mediated through the angiotensin-converting enzyme 2 (ACE2) receptor, highly expressed on alveolar epithelial cells and variably present in tissues such as the oral mucosa, myocardium, kidneys, and liver. The pathogenesis of COVID-19 is characterized by dysregulated immune responses including cytokine storm, chemokine overproduction, and leukocyte influx, which drive multi-organ involvement and disease severity (Tang et al., 2020; Al et al., 2020; Brodin, 2021; Kgatle et al., 2021; Rejano-Gordillo et al., 2022).

Recent studies have implicated the aryl hydrocarbon receptor (AHR), a ligand-activated transcription factor, as a critical modulator in SARS-CoV-2 infection. AHR has historically been recognized for its role in xenobiotic metabolism, but growing evidence supports its broader function in immunoregulation, cellular differentiation, and inflammatory signalling (Rejano-Gordillo et al., 2022). Viral pathogens such as Zika virus (ZIKV) and Dengue virus (DENV) exploit AHR signalling to enhance replication and evade host defences, and emerging data suggest a similar mechanism in SARS-CoV-2 infection (Hu et al., 2023; Giovannoni et al., 2021; Shi et al., 2023).

Notably, SARS-CoV-2 activates AHR through an indoleamine 2,3-dioxygenase 1 (IDO1)-independent pathway that bypasses kynurenine accumulation. This leads to transcriptional upregulation of downstream effectors such as TiPARP, IL-10, IL-1β, and tumour necrosis factor-alpha (TNF-α), key contributors to immune dysregulation and inflammation (Mazari et al., 2025; Giovannoni and Quintana, 2021; Turski et al., 2020). In COVID-19, AHR activation has been associated with impaired interferon signalling, altered ACE2 expression, and sustained viral proliferation (Shi et al., 2023).

Beyond its immunological role, AHR influences gene expression via epigenetic modifications including chromatin remodelling, microRNA regulation, histone acetylation, and DNA methylation (Rothhammer and Quintana, 2019; S et al., 2014). These mechanisms collectively shape immune responses and contribute to disease progression (Cannon et al., 2021). In individuals with comorbidities such as obesity and diabetes, AHR overactivation further exacerbates metabolic dysfunction, endothelial injury, and thrombotic risk.

Emerging SARS-CoV-2 variants of concern (VOCs), such as LP.8.1 and XEC, continue to undermine vaccine efficacy and challenge existing therapeutic strategies. Despite genetic divergence, these variants consistently exploit the AHR signalling pathway to support viral replication and immune evasion, indicating that AHR plays a conserved role across variant lineages. The structural integrity of key AHR domains, including PAS-B and transactivation regions, further supports its relevance as a stable target for host-directed therapy (Del Sorbo et al., 2025; Mambelli et al., 2025; Roederer et al., 2024). While current vaccines do not directly modulate AHR activation, they may influence AHR-related pathways indirectly by tempering systemic inflammation and reducing cytokine overload, which are known contributors to AHR signalling. Importantly, AHR activation patterns appear consistent across variants, suggesting that its therapeutic targeting remains viable irrespective of mutational changes in the virus. In relation to 2025s landscape of variant-driven immune escape, AHR modulation offers a complementary approach to enhance immune control and reduce disease severity, particularly in individuals with reduced vaccine responsiveness or comorbid conditions.

The significance of this review lies in its timely focus on AHR as a multifaceted regulator of disease severity, particularly in individuals with underlying metabolic and inflammatory comorbidities. With the rise of immune-evasive variants and limited efficacy of conventional therapies in certain populations, understanding AHR-driven pathways offers a strategic entry point for host-targeted interventions. By elucidating the molecular mechanisms of AHR activation in SARS-CoV-2 infection, this review builds a compelling case for therapeutic modulation of AHR as a precision medicine approach against COVID-19.

## Structural components and signalling of AHR

**FIGURE 1:**
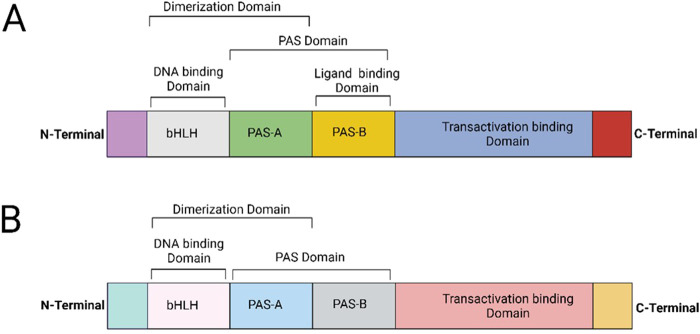
**(A)** The structure of the AHR. An amine N terminal, a bHLH, and a central PAS domain with two repeats for ligand binding: PAS-A and PAS-B are highlighted. Near the C terminal end, there is a transactivation domain (TAD). The bHLH enhances AHR’s binding to the promoter regions in the DNA of its target genes, while the PAS-B domain is involved in ligand binding to AHR. **(B)** In comparison, the structure of ARNT contains a bHLH that binds to the promoter sequence in the DNA of its target genes. However, the PAS-B domain in ARNT does not bind any ligands.

AHR belongs to the basic helix-loop-helix (bHLH)/PER-ARNT-SIM (PAS) superfamily of proteins (Rejano-Gordillo et al., 2022). It features a bHLH domain near the N-terminal, which aids in binding AHR to the promoter regions of targeted genes and supports protein dimerization (Hu et al., 2023; Biagioli et al., 2021; Guarnieri, 2022). The PAS domain provides structural integrity and ligand binding, facilitating interactions with the aryl hydrocarbon nuclear translocator (ARNT) and other ligands (Turski et al., 2020; Nebert, 2017). AHR targets genes through consensus regions known as aryl hydrocarbon response elements (AHRE) and dioxin/xenobiotic response elements (DRE/XRE) (Nebert, 2017; Dong and Perdew, 2020). The C-terminal region contains the transactivation domain, which interacts with co-activators or co-repressors to regulate transcription (Turski et al., 2020; Nebert, 2017; Dong and Perdew, 2020; Xue et al., 2018) (Figure 1).

AHR regulates various physiological processes, including immune responses, metabolism, and cellular differentiation. AHR activation is influenced by a wide range of ligands, with exogenous ligands often causing toxicological effects, while endogenous ligands maintain physiological balance and regulate immune functions (Nebert, 2017; Barturen et al., 2022; Bradic et al., 2022). Exogenous ligands, such as environmental contaminants like polycyclic aromatic hydrocarbons (PAHs) and halogenated aromatic hydrocarbons (HAHs), as well as dietary compounds like curcumin, indigo, and indirubin, can activate AHR, often leading to toxic effects such as immunotoxicity, cardiotoxicity, and hepatotoxicity (Ashida et al., 2008). These effects arise from AHR activation by xenobiotics, resulting in the expression of cytokines and chemokines that disrupt normal immune cell function.

Endogenous ligands, naturally occurring within the body, include tryptophan (TRP) metabolites like kynurenine (KYN) and kynurenic acid (KYNA), indigo and indirubin, and 6-formylindolo (3,2-b)carbazole (FICZ) (Nebert, 2017). These endogenous ligands play crucial roles in maintaining normal cellular functions, including immune responses and cellular differentiation (Nebert, 2017; Balnis et al., 2023; Konigsberg et al., 2021). For example, AHR activation by FICZ promotes the development of T helper 17 (Th17) cells, which are involved in inflammatory responses (Sládeková et al., 2023). KYN, a significant endogenous ligand for AHR, plays a crucial role in SARS-CoV-2-induced AHR activation. It is produced through the initial and rate-limiting step of the KYN pathway, the primary route for tryptophan catabolism in the body, leading to various immunomodulatory effects.

With the emergence of the COVID-19 pandemic, studies suggest that AHR activation may contribute pro-inflammatory responses, potentially exacerbating cytokine storm in SARS-CoV-2 infection (Bradic et al., 2022; Torti et al., 2021). This activation could explain alteration in tumor necrosis factors (TNFs), interferons (IFNs), interleukin (ILs), chemokines, and acute-phase proteins such as ferritin, D-dimer, transaminase, bilirubin and C-reactive protein (CRP) (Del Sorbo et al., 2025). Elevated levels of IL-1β, IL-6, IL-8 and TNF-α induced by AHR and indoleamine-2,3-dioxygenase 1 (IDO1) may lead to extensive tissue damage and severe disease progression (Konigsberg et al., 2021; Xiao and Vermund, 2024).

**FIGURE 2:**
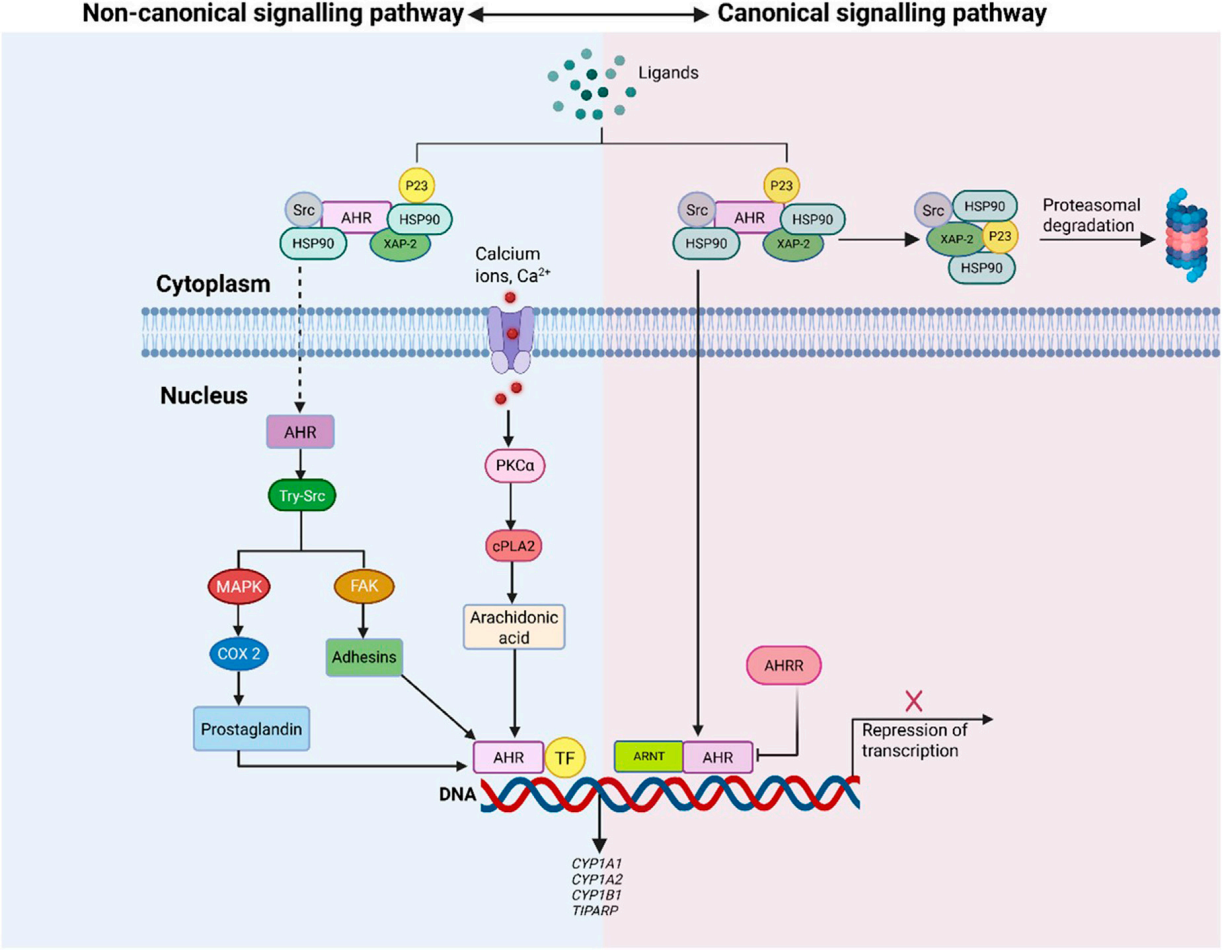
Overview of Canonical vs Non-Canonical AHR Signalling Pathways. The figure compares AHR activation via canonical and non-canonical signalling. In the canonical pathway, ligand-bound AHR translocates to the nucleus, dimerizes with ARNT, and regulates target gene transcription through XRE binding. The non-canonical pathway involves AHR activation by diverse ligands, initiating secondary signalling cascades (e.g., MAPK, calcium-mediated PKCα) and modulating gene expression via interactions with transcription factors beyond XRE targets.

**FIGURE 3:**
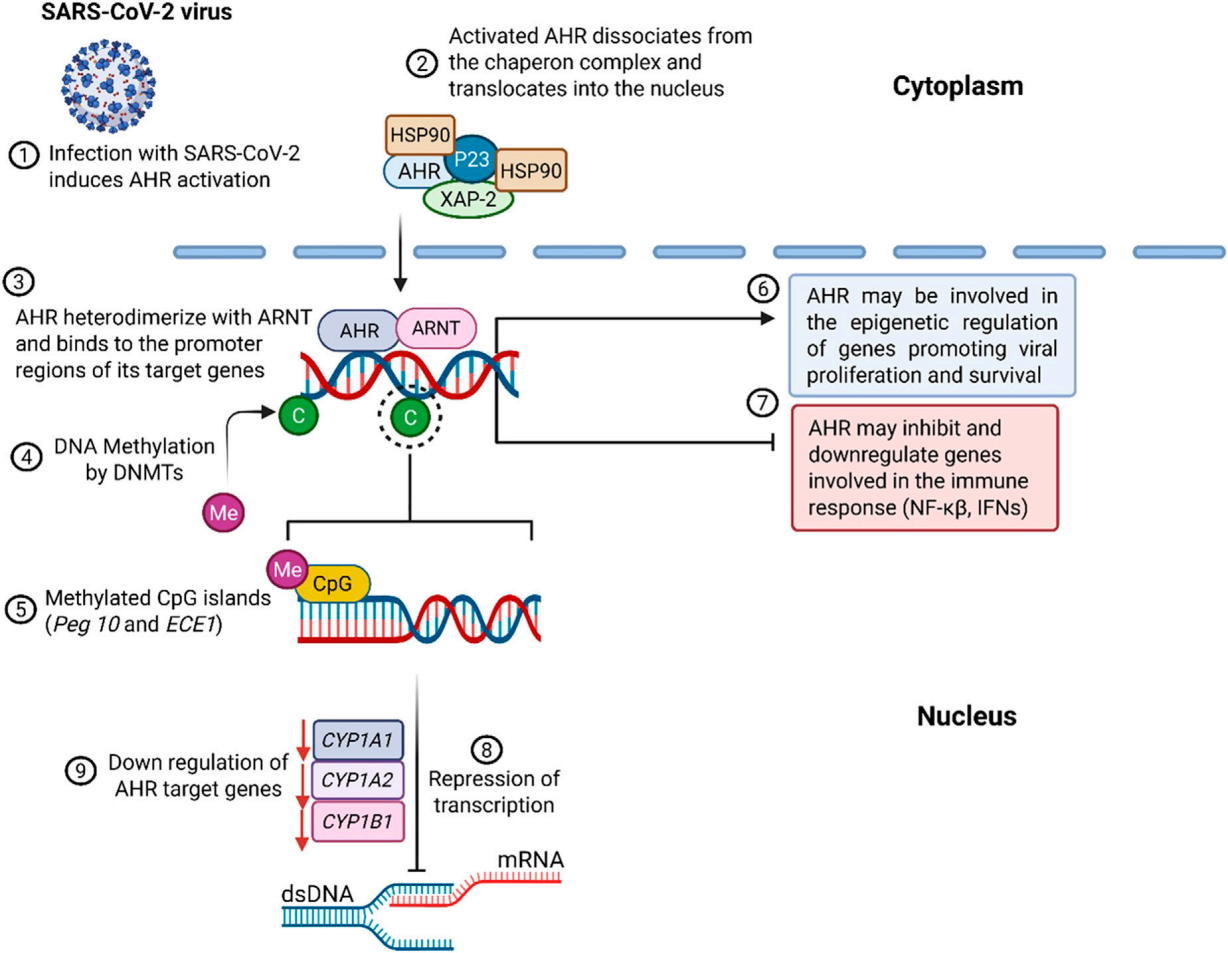
Infection with SARS-CoV-2 triggers the activation of AHR. Once activated, AHR translocates to the nucleus, where it forms a heterodimer with ARNT and binds to the promoter region of its target genes. Epigenetic regulation of AHR, particularly through DNA methylation catalyzed by DNMTs, can lead to the upregulation of genes (e.g. Peg10 and ECE1) involved in innate immune and inflammatory responses. AHR’s epigenetic regulation can also inhibit transcription and reduce the expression of genes involved in the transcription process.

Under basal conditions, AHR is sequestered in the cytoplasm, stabilized by a chaperone complex comprising heat shock protein 90 (HSP90), co-chaperone p23 and hepatitis b virus X associated protein 2 (XAP2) (Sládeková et al., 2023). This complex preserves receptor conformation and prevents premature degradation. Canonical activation is initiated when specific ligands bind AHR, inducing a conformational change that exposes its nuclear localization signal (NLS), as described in Figure 2. This enables AHR’s translocation into the nucleus, where it dimerizes with ARNT to form the functional AHR/ARNT complex (Figure 3). This complex binds xenobiotic response elements (XREs) on target gene promoters, regulating transcription of immunomodulatory and metabolic genes such as *CYP1A1*, *CYP1A2*, and *CYP1B1*, which metabolize AHR ligands and facilitate signal termination (Sládeková et al., 2023; Torti et al., 2021).

The non-canonical pathway engages a broader array of cellular mechanisms and ligand types, including calcium ions and environmental toxins like 2,3,7,8 -tetrachlorodibenzo [p] dioxin (TCDD) (Hu et al., 2023). Calcium influx elevates PKCα activity, triggering cytosolic phosphate A 2 (cPLA2) phosphorylation and arachidonic acid production. In parallel, TCDD-bound AHR activates tyrosine-Src (tyr-Src) and mitogen activated protein kinase (MAPK) signalling cascades, which regulate focal adhesion kinase (FAK) and adhesion molecules. MAPK also enhances transcription of cyclooxygenase 2 (COX2), supporting prostaglandin synthesis and propagating inflammatory signals (Torti et al., 2021).

Additionally, non-canonical AHR interacts directly with transcriptional regulators such as nuclear factor kappa beta (NF-κβ), activator protein-1 (AP-1) and oestrogen receptor (ER), and signal transducers and activators of transcription (STATs), modulating their activity via transactivation, transrepression, or direct protein interactions (Sládeková et al., 2023). These cross-talk mechanisms integrate AHR into broader inflammatory and immunological circuits, establishing it as a multifaceted signalling hub.

## The role of AHR in SARS-COV-2 immune modulation and viral replication

The AHR plays a central role in regulating host immune responses during viral infections, including those caused by coronaviruses such as SARS-CoV-2. Mechanistically, AHR orchestrates gene expression via multiple epigenetic pathways, chromatin remodelling, microRNA regulation, histone acetylation, and DNA methylation, which collectively influence immune homeostasis and disease progression (reviewed in (Giovannoni and Quintana, 2021)). Viral activation of AHR is recognized as a strategic immune evasion tactic that facilitates replication and exacerbates pathogenesis (Figure 3). In regard to COVID-19, SARS-CoV-2 triggers AHR signalling upon ACE2-mediated cellular entry, leading to mucin hypersecretion and suppression of key antiviral pathways, particularly type I interferon (IFN-I) and NF-κB signalling, thereby promoting viral persistence and respiratory pathology (9, 31, Figure 3).

AHR functions as a proviral factor across several viruses, including ZIKV, DENV, and HSV-1, with inhibition studies demonstrating reduced viral loads and restored immune signalling (Anderson et al., 2021; Chilosi et al., 2022). Through its transcriptional networks, AHR engages host and viral genomic elements, regulating effector proteins such as TiPARP, which supports coronavirus replication (Hu et al., 2023). Immunologically, AHR modulates CD4^+^ T cell subset polarization, influencing the Th17, Treg, and Tr1 balance. Its control over FOXP3, the master transcription factor of Tregs, exemplifies its epigenetic reach (Badawy, 2023; Dehhaghi et al., 2024). Dysregulation within the Treg/Th17 axis is associated with hyperinflammatory states, including cytokine storm and multi-organ failure in severe COVID-19 cases (Dehhaghi et al., 2024; Balaton et al., 2015).

Beyond general immune modulation, AHR-related epigenetic factors intersect with sex-based differences in disease outcomes. Males consistently exhibit higher ICU admissions and mortality; a trend partly linked to X-chromosome inactivation (XCI). XCI is an epigenetic process that allows females to selectively express immune-related genes from the inactivated X chromosome, enhancing immune responsiveness and providing protection against X-linked disorders (Balaton et al., 2015; Gendrel et al., 2012; Harper, 2011). Coronaviruses have been shown to exploit host epigenetic machinery, including DNA methyltransferases (DNMTs) and histone modifiers, to repress antiviral genes and sustain viral persistence, reviewed in (Giovannoni and Quintana, 2021), and illustrated in Figure 3. SARS-CoV-2 infection has been associated with differential methylation of *Peg10* and *ECE1*. *Peg10* is a paternally imprinted gene that plays a critical role in cell proliferation, survival, and oncogenic pathways (Ono et al., 2006). In contrast, ECE1 encodes *endothelin-converting enzyme-1*, which regulates vascular tone and has been associated with cardiac stress and injury (Shimada et al., 1995). Epigenetic modulation of these genes during COVID-19 highlights a potential mechanism underlying both long-term proliferative disorders and cardiovascular complications observed in patients (Figure 3) (Ono et al., 2006; Shimada et al., 1995).

Comparative analyses across SARS-CoV-1, MERS-CoV, and animal coronaviruses reveal conserved AHR activation profiles and transcriptional remodelling, particularly through downstream targets such as *CYP1A1* and *CYP1B1* (Cannon et al., 2021; Seo and Kwon, 2023; Shadboorestan et al., 2023). These viruses hijack cellular stress and xenobiotic pathways to amplify AHR activity, further dampening pro-inflammatory cytokine production and compromising immune defence (Giovannoni et al., 2021). Variant-specific modulation of AHR has also been observed (Guarnieri, 2022). For instance, the Delta variant elevates IL-6 and IFN-γ production, upregulating IDO1 and enhancing kynurenine-mediated AHR signalling. In contrast, Omicron induces a muted cytokine response, potentially attenuating AHR-driven suppression (Barh et al., 2023; Korobova et al., 2022; Kr et al., 2025; Rangchaikul and Venketaraman, 2021; Shahbaz et al., 2023). Despite these variant differences, data on strain-specific interactions with the IDO1-KYN-AHR axis remain limited, warranting further investigation.

AHR’s role in viral pathogenesis extends beyond coronaviruses. In infections like human cytomegalovirus (HCMV), HIV, and ZIKV, pathogens manipulate TRP metabolism to produce kynurenine and other endogenous AHR ligands, establishing a metabolic-immune interface conducive to viral latency and immune suppression (Kgatle et al., 2021; Anderson et al., 2021; Balaton et al., 2015). Elevated kynurenine levels correlate with high viral burden and reactivation risks, especially in HCMV-infected cells (Giovannoni et al., 2021). AHR also influences the crosstalk between innate and adaptive immunity, impacting replication kinetics and immune tolerance (Kgatle et al., 2021). By directly engaging both host and viral genetic elements, AHR perpetuates viral replication and immune dysregulation (Anderson et al., 2021).

Taken together, the convergence of AHR signalling, sex-specific epigenetic dynamics, and viral reprogramming mechanisms positions AHR as a critical immunomodulatory node exploited by diverse viruses. Its inhibition not only holds promise in restoring antiviral defence but also presents a targeted therapeutic avenue to mitigate disease severity across multiple viral infections.

## The role of IDO/KYN in AHR activation and COVID-19 pathogenesis

KYN, a key metabolite of TRP degradation, is a potent endogenous ligand of the AHR (Seo and Kwon, 2023; Shadboorestan et al., 2023; Solvay et al., 2023). Upon activation, AHR orchestrates a cascade of immune-modulatory effects, including suppression of antiviral signalling and promotion of an immunosuppressive environment. This pathogen-exploitable mechanism is not exclusive to SARS-CoV-2; several viruses leverage the IDO1-KYN-AHR axis to facilitate infection and evade host immune defences (Chilosi et al., 2022). IDO1 is a member of the IDO enzymes, which also includes IDO2.

**FIGURE 4:**
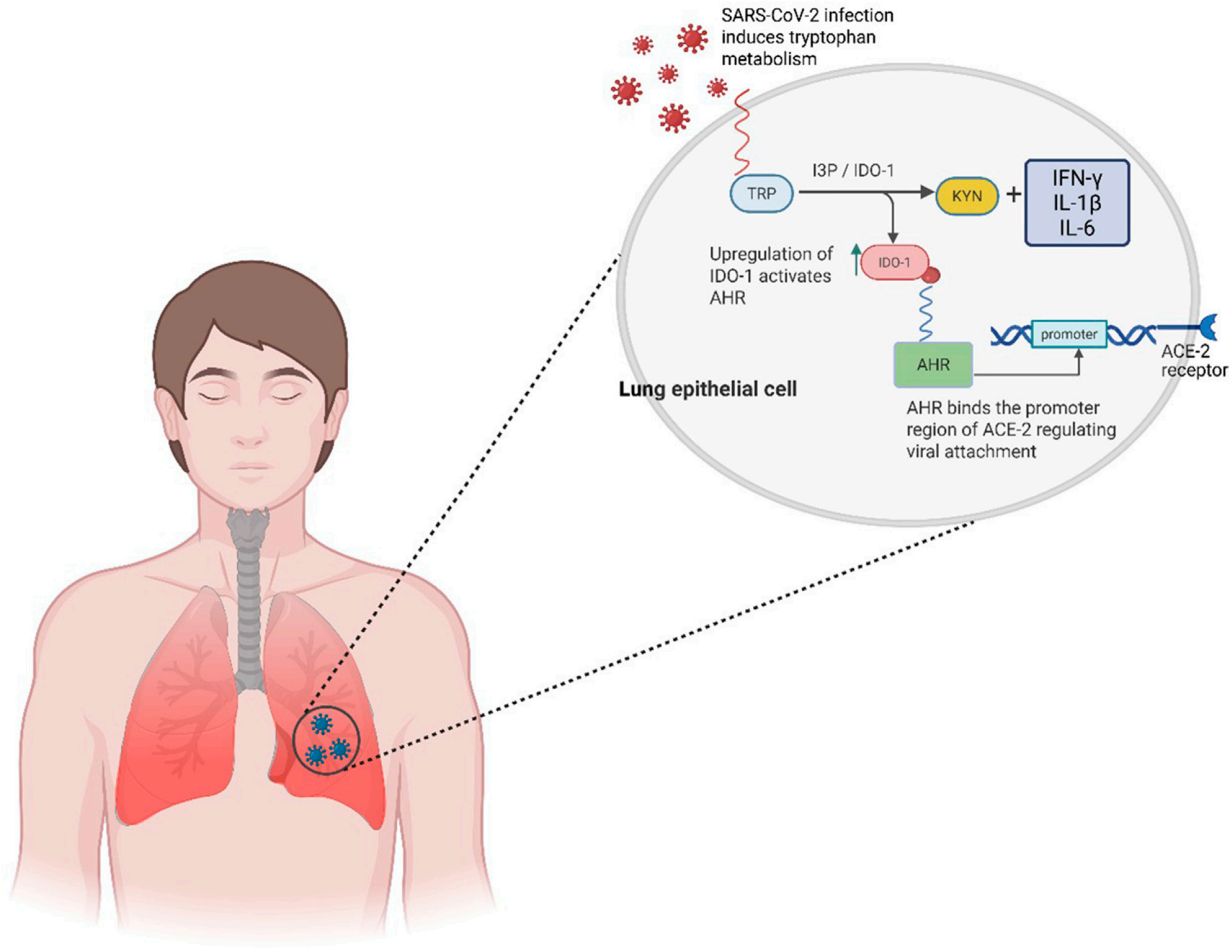
The role of IDO/KYN in AHR activation. When infected with SARS-CoV-2, tryptophan metabolism is triggered, leading to the production of kynurenine via an intermediate called IDO1. The upregulation of IDO1, a rate-limiting molecule, acts as an endogenous ligand that activates AHR. The activated AHR then binds to the promoter regions of the ACE2 receptor, regulating its function and facilitating viral attachment and entry into the cells.

During COVID-19, AHR activation contributes broadly to disease progression, modulating immune responses, vascular integrity, and neurological function. The enzyme IDO1, which initiates TRP catabolism, is significantly upregulated during SARS-CoV-2 infection. Its isoform, IDO2, is also expressed, with IDO1 linked to early-to-mild pneumonia and IDO2 prevalent in more severe and fatal presentations (Giovannoni et al., 2021). COVID-19–induced inflammation elevates key cytokines such as IFN-γ, IL-1β, and IL-6, which potently induce IDO1 expression in immune cells including macrophages, fibroblasts, and dendritic cells (Figure 4) (Anderson et al., 2021; Chilosi et al., 2022; Dehhaghi et al., 2024).

IDO1 catalyses the conversion of TRP to KYN, which is subsequently metabolized by kynurenine aminotransferase (KAT) into kynurenic acid (KYNA), both recognized AHR agonists (Chilosi et al., 2022). TRP, an essential amino acid with critical roles in protein synthesis, immune regulation, and neurological function, becomes increasingly depleted under these inflammatory conditions, amplifying downstream AHR signalling (Giovannoni et al., 2021) (Figure 4).

IDO1 is highly expressed in macrophages, microglia, neuronal cells, and mucosal tissues such as the placenta and gut (Dehhaghi et al., 2024). Its role in TRP breakdown along the KYN pathway generates metabolites with profound immunological effects, including dampening of pro-inflammatory cytokines and alteration of immune cell differentiation (Anderson et al., 2021; Badawy, 2023). In regard to COVID-19, this metabolic-immune interplay is increasingly recognized as a driver of pathogenesis (Giovannoni and Quintana, 2021; Bowler et al., 2022; Salem et al., 2023) (Figure 4).

Notably, SARS-CoV-2 and related coronaviruses may manipulate AHR activation indirectly through modulation of Ti-PARP and cytokine networks via the IDO1-KYN pathway, sometimes operating independently of AHR itself (Anderson et al., 2021; Dehhaghi et al., 2024). Initially quiescent during mild or subclinical infection, the IDO1-KYN axis becomes hyperactivated as inflammation escalates, leading to Systemic AHR Activation Syndrome (SAAS) and sustained immune dysfunction (Anderson et al., 2021; Badawy, 2023). This dysregulation impairs natural killer (NK) cell function, suppresses CD8^+^ T cell responses, and downregulates MHC-II expression, contributing to immune cell exhaustion and viral persistence (Badawy, 2023).

Elevated KYN and AHR activation are also associated with melatonin deficiency, due in part to SARS-CoV-2-mediated suppression of ACE2, which impairs TRP absorption in the gut (Murray and Perdew, 2020). This metabolic shift further exacerbates immunosuppression and may increase disease severity. Additionally, lipopolysaccharide (LPS)-induced inflammation serves as a secondary trigger for AHR activation, compounding the severity of COVID-19 (Chen Q. et al., 2023; Ramasamy and Subbian, 2021; Sen, 2022). Beyond its transcriptional role, nuclear AHR may act as an E3 ubiquitin ligase, mediating chromatin remodelling and contributing to epigenetic dysfunction, further amplifying inflammatory and immunosuppressive signals during advanced disease stages.

The immunomodulatory function of the IDO1-KYN-AHR axis in COVID-19 has prompted interest in IDO1 inhibition as a potential therapeutic strategy. While IDO1 inhibitors are well-characterized in oncology and infectious diseases, their application in COVID-19 is still emerging. Preclinical evidence supports the potential efficacy of IDO1 inhibition: *in vitro* studies using THP-1 and RAW264.7 cell lines exposed to cytokine storm conditions showed that IDO1 inhibitors such as 1-methyl-tryptophan (1-MT) and Y103 effectively suppressed IDO1 activity, reduced AHR expression, and downregulated key inflammatory mediators such as CYP1A1 (Badawy, 2023). Furthermore, dexamethasone, a clinical agent used in COVID-19 treatment, exerts partial inhibitory effects on IDO1 and AHR through glucocorticoid receptor-mediated pathways, contributing to reduced cytokine storm severity and enhanced viral clearance (Badawy, 2023).

Although these findings highlight mechanistic rationale for IDO1 inhibition in SARS-CoV-2 infection, clinical studies directly assessing IDO1-specific inhibitors in COVID-19 populations remain limited. The dual role of IDO1 in promoting immune tolerance and suppressing excessive inflammation poses challenges in therapeutic targeting, particularly given its tissue-specific expression and disease phase-dependent activity. Nonetheless, modulation of this pathway continues to be explored, and may offer adjunctive benefits when combined with anti-inflammatory or antiviral therapies.

## The activation of AHR in COVID-19-known comorbidities

The activation of the AHR plays a pivotal role in modulating immune, inflammatory, and metabolic pathways during COVID-19, particularly in individuals with pre-existing comorbidities. Across these conditions, AHR signalling has been shown to intensify disease severity by promoting immune suppression, chronic inflammation, and physiological dysfunction.

AHR is activated by SARS-CoV-2 itself as well as by environmental ligands such as dioxins and TRP-derived metabolites. This activation leads to the upregulation of immunosuppressive cytokines, notably IL-6, IL-10, and TGF-β, which contribute to persistent inflammation and impaired antiviral immunity. Elevated levels of IL-6 and TGF-β have been correlated with severe lung inflammation and fibrosis, as well as dysregulation of the kynurenine pathway (KP), a TRP metabolic pathway consistently disturbed in COVID-19 patients (Chatterjee and Maparu, 2025; Hu et al., 2021; Moura-Alves et al., 2014; Ragab et al., 2020; Thomas et al., 2020).

Emerging data suggest that AHR-related effects may persist beyond acute infection, contributing to post-viral sequelae characteristic of long COVID (Singh et al., 2022). In individuals recovering from mild to severe COVID-19, the KP remains markedly upregulated, particularly in cases where mild cognitive deficits are observed. Key KP metabolites, including 3-hydroxyanthranilic acid, kynurenine, and quinolinic acid, are known endogenous AHR ligands and have been associated with sustained AHR activation in peripheral circulation. This chronic engagement of AHR signalling may perpetuate neuroinflammation, immunosuppression, and metabolic dysregulation, thereby contributing to symptoms such as brain fog, fatigue, and cognitive decline seen in long COVID (Chatterjee and Maparu, 2025; Hu et al., 2021; Moura-Alves et al., 2014; Ragab et al., 2020; Thomas et al., 2020; Singh et al., 2022). These findings underscore the possibility that SARS-CoV-2-induced metabolic remodelling through the KP can result in persistent AHR-driven transcriptional programs that outlast viral clearance (Singh et al., 2022).

A study by Anderson et al. (2020) highlighted that pro-inflammatory cytokines can perpetuate AHR activation, which in turn suppresses antiviral responses and exhausts immune cells, worsening SARS-CoV-2 pathogenesis. Gupta et al. (2025) reported that chronic inflammation and immune dysregulation driven by AHR activity may account for the persistence of long COVID symptoms (Gupta et al., 2025). AHR’s influence on T-helper 17 (Th17) cell differentiation and IL-22 secretion, as described by Moura-Alves et al. (2014), may further exacerbate respiratory inflammation and cytokine storm events in COVID-19 patients. Furthermore, AHR suppresses IFN-α/β responses, key antiviral defences, thereby prolonging viral replication and increasing disease severity (Moura-Alves et al., 2014).

In chronic lung diseases such as chronic obstructive pulmonary disease (COPD) and asthma, AHR activation worsens respiratory function through enhanced mucus production and airway remodelling. Chiba et al. (2011) demonstrated that AHR signalling in airway epithelial cells upregulates mucin genes MUC5AC and MUC5B, contributing to mucus hypersecretion (Chiba et al., 2011). In COPD, AHR promotes goblet cell differentiation and airway obstruction, conditions that heighten the risk of COVID-19-related respiratory failure (Bornstein et al., 2021; Steenblock et al., 2021).

Heightened AHR activity also facilitates fibrotic signalling through TGF-β and IL-22, increasing susceptibility to long-term pulmonary fibrosis. Persistent AHR activation drives epithelial-to-mesenchymal transition (EMT), a hallmark of fibrotic remodelling (Simonian et al., 2010). AHR-mediated induction of TGF-β1 has been implicated in post-viral fibrosis, and studies show that AHR inhibition can reduce fibrotic gene expression in lung tissues, supporting its potential as a therapeutic target (Alfaro et al., 2024).

AHR signalling is markedly altered in individuals with metabolic disorders such as obesity, diabetes, and hypertension, conditions closely linked to severe COVID-19 outcomes. In obesity, dietary and microbial metabolites can hyperactivate AHR, leading to insulin resistance and chronic low-grade inflammation (Bock, 2021; da Silva et al., 2022). This disrupts adipose tissue homeostasis and elevates pro-inflammatory cytokines such as IL-6 and TNF-α, compounding metabolic dysfunction (Bornstein et al., 2021; Steenblock et al., 2021).

AHR’s role in glucose metabolism is especially critical during SARS-CoV-2 infection. Recent studies reveal that AHR activation in pancreatic β-cells impairs insulin secretion, increasing the risk of hyperglycaemia (Jedrzejak et al., 2022). Elevated AHR activity also contributes to vascular injury, heightening the risk of thrombotic complications, stroke, and myocardial injury via mechanisms involving oxidative stress and endothelial dysfunction (Bornstein et al., 2021).

In type 2 diabetes mellitus (T2DM), AHR activation can interfere with insulin receptor signalling and amplify vascular inflammation. This enhances susceptibility to severe COVID-19 complications including diabetic ketoacidosis and multi-organ failure (Memon and Abdelalim, 2021). In hypertensive individuals, AHR contributes to cardiovascular risk by suppressing ACE2, a key regulator of the renin-angiotensin system (RAS). The downregulation of ACE2 leads to increased angiotensin II levels, promoting inflammation and vasoconstriction (Wu et al., 2021). AHR-targeted therapies, including the use of antagonists, may offer therapeutic promise for mitigating cardiometabolic complications in COVID-19.

Healey et al. (2024) reported that AHR activation alters immune cell populations in both the lungs and bone marrow of murine coronavirus-infected models, highlighting AHR’s persistent role beyond acute infection. The study suggests that long COVID, characterized by sustained immune dysregulation, may be driven in part by prolonged AHR signalling. These insights reveal AHR as a key regulator of host-pathogen interactions, with far-reaching implications for post-viral recovery and therapeutic intervention (Healey et al., 2024).

## Potential therapeutic agents targeting AHR in COVID-19

The AHR, described as an environmental sensor and transcription factor, responds to xenobiotics, dietary metabolites, microbial byproducts, and viral components to modulate cellular homeostasis and immune signalling. In the context of COVID-19, AHR represents a promising therapeutic target for modulating aberrant inflammatory responses. Agonists may suppress cytokine storms and hyperinflammation, whereas antagonists could reverse virus-induced immune suppression and fibrosis (Grishanova and Perepechaeva, 2024; Xu et al., 2024).

Dietary ligands such as indole-3-carbinol (I3C) and diindolylmethane (DIM), derived from cruciferous vegetables, activate AHR to induce anti-inflammatory cytokines and dampen immune overactivation (Bahman et al., 2024; Huang et al., 2023). Endogenous ligands from gut microbiota further contribute to immune homeostasis, suggesting that dietary interventions and probiotics could restore immunological balance (Bahman et al., 2024). Small-molecule modulators like Tapinarof, with established anti-inflammatory properties, and synthetic compounds such as Furans, PCB153, Benzo(a)pyrene (BaP), and Benz(a)anthracene (BA) are under investigation for attenuating lung injury and promoting regulatory immune responses (Sládeková et al., 2023; Chen Y. et al., 2023). These modulators are described in Table 1.

**TABLE 1:** Selected key agents of AHR used in therapeutics that may have effect in COVID-19.

AHR agent	Agonist/antagonist	Stage of testing	Benefits	Risks	Ref
Tapinarof	agonist	Phase 2,3 trial	Anti-inflammatory, anti-proliferative, acts through immune regulation, skin barrier restoration, oxidative stress	Gastroenteritis, nasopharyngitis	Igarashi et al. (2024), Igarashi et al. (2025), Santini et al. (2025)
BAY2416964	antagonist	Preclinical, phase 1	Blocks AHR activation through the KYN, exerts immunosuppressive effects of AHR	Nause, fatigue	Dumbrava et al. (2023), Papadopoulos et al. (2023)
GNF351	antagonist	FDA approved	Inhibits both the genomic and non-genomic activation of AHR	Poor absorption and extensive metabolism	Fang et al. (2014), Ondrová et al. (2023)
BaP	antagonist	_	Induces apoptosis and CYP1A1 activity during AHR activation	Induces cardiac and mutagenic effects of damaged DNA	Zou et al. (2024)

Biomarker discovery and drug screening for AHR-targeted therapies are increasingly supported by high-throughput technologies and artificial intelligence. Various agents are currently being developed not only for systemic treatment but also for use as companion diagnostics, particularly in inflammatory skin conditions like psoriasis (Haarmann-Stemmann et al., 2025). These efforts highlight AHR’s diagnostic and therapeutic versatility.

Nevertheless, prolonged or excessive AHR activation poses risks, including immune suppression, viral persistence, and tissue fibrosis, concerns particularly relevant in long COVID-19 (Torti et al., 2021; Giovannoni et al., 2020; van den Boga et al., 2015). Selective AHR antagonists such as CH-223191 and GNF351 have demonstrated potential to restore IFN-1 responses, reduce fibrosis, and rebalance mucosal immunity (Ghiboub et al., 2020). Additionally, dietary strategies aimed at modulating tryptophan metabolism and reducing pro-inflammatory AHR ligands offer complementary approaches (Riaz et al., 2022).

Safety remains a critical challenge for chronic AHR modulation due to its complex role in regulating immune balance and detoxification processes (Cannon et al., 2021; Zhu et al., 2014). Depending on ligand specificity and metabolism, AHR activation can either suppress or exacerbate autoimmune conditions such as lupus, rheumatoid arthritis, multiple sclerosis, and atopic dermatitis, primarily through its effects on Th17 and Treg cell dynamics, reviewed in Zhu et al. (2014). While therapeutic agonists like TCDD, ITE, curcumin, and DIM show promise in promoting immunologic tolerance and reducing inflammation, rapidly metabolized ligands like FICZ may aggravate disease progression. Additionally, prolonged AHR modulation may impair cytochrome P450-mediated detoxification, increasing vulnerability to environmental toxins (reviewed in Zhu et al., 2014). Toxicogenomic profiling and transcriptomic analysis are valuable tools for identifying ligand-specific toxicities and differentiating transient from sustained AHR activation effects. Overall, therapeutic strategies must differentiate between protective and harmful ligands, considering both immune and metabolic outcomes.

Collectively, these findings underscore the therapeutic promise of targeting AHR in COVID-19. Continued research into ligand selectivity, delivery mechanisms, and integrated omics-based screening may guide the development of precise, safe, and effective interventions for mitigating inflammation and improving outcomes in COVID-19 and its associated complications.

## Conclusion and future perspectives

The AHR continues to garner significant attention due to its multifaceted transcriptional functions, ranging from xenobiotic metabolism to regulation of immune and inflammatory signalling pathways. Evidence suggests that AHR activation during coronavirus infection contributes to viral replication, immune evasion, and worsened disease severity, particularly in individuals with underlying conditions such as diabetes, hypertension, and respiratory disorders. Transcriptional regulation of AHR further amplifies inflammation and metabolic dysfunction, positioning AHR as a key molecular mediator in COVID-19 pathogenesis.

In response to its diverse biological roles, recent advances have focused on developing AHR-based biomarkers and companion diagnostics. Artificial intelligence tools now enable efficient screening and modelling of ligand–AHR interactions, accelerating therapeutic discovery. Tapinarof, a clinically explored AHR modulator, exemplifies this translational progress, serving as both a topical agent and a diagnostic companion in psoriasis immunotherapy, with potential applications in COVID-19-related immune regulation.

Despite these promising developments, further investigation is essential to clarify the precise signalling pathways through which AHR contributes to viral propagation and immune imbalance. Deciphering its genetic and epigenetic regulation, as well as identifying interacting regulatory proteins, may pave the way for interventions that selectively suppress pathological AHR activity while preserving its physiological functions.

Current research into AHR antagonists such as CH-223191 and GNF351 shows potential in reversing inflammation and metabolic derangements associated with COVID-19. Complementary approaches, including dietary modulation and probiotic-driven regulation of tryptophan metabolism, may help restore immune homeostasis. These emerging therapeutic and diagnostic strategies underscore the importance of continued exploration of AHR biology in COVID-19 and other inflammatory diseases. Advancing this field could significantly improve clinical outcomes and reduce long-term complications.

## Abbreviations

ACE2, Angiotensin converting enzyme 2; AHR, Aryl hydrocarbon receptor; ARNT, Aryl hydrocarbon receptor nuclear translocator; bHLH, Basic Helix Loop Helix; COVID-19, Coronavirus Disease 2019; EMT, Epithelial-to-mesenchymal transition; FICZ, 6-formylindolo (3,2-b) carbazole (FICZ); IDO1, Indoleamine-2, 3-dioxygenase 1; IL, Interleukin; HAH, Halogenated aromatic hydrocarbons; KYNA, Kynurenic acid; PAH, Polycyclic aromatic hydrocarbons; RAS, Renin-angiotensin system; T2DM, Type 2 diabetes mellitus; TGF, Tumour growth factor; TNF-α, Tumour necrosis factor alpha; TRP, Tryptophan.
